# Electric Lighting for Hospitals

**Published:** 1894-11-03

**Authors:** 


### ELECTRIC LIGHTING FOR HOSPITALS.

Mr. F. W. Reuss writes: I have just read an extract in the *Echo* from your paper "Electric Light in Hospitals." Will you allow me to inform you that the Guardians of the Dewsbury Union, of which I have the honour to be a member, have the installation of the electric light now in progress, not only for their model new infirmary, but for the workhouse, children's homes, &c., as well. More than this, throughout the whole of the buildings gas is provided as well, all brackets and chandeliers, &c., are on the duplex system, a switch and a tap are close together, the one to turn on the electric light, the other the gas in case of the electric light failing. Our infirmary and children's homes (the latter under foster-mothers, twelve being in every house, boys, girls, and infants, altogether away from the inmates of the house) are recommended by the Local Government Board inspectors as model institutions, which ought to be visited by Guardians of other unions who have to make fresh provisions. The cost of installation of the electric light, about 500 lights, is about £2,000, exclusive of boilers, which the Guardians had already provided for heating purposes.

[It must not be imagined from this letter that a hospital provided with electric light requires to be fitted with gas also. Electric installations have reached a perfection which makes this quite unnecessary.—Ed. *T. H.*]

